# Phenotypic and molecular basis of *SIX1* variants linked to non-syndromic deafness and atypical branchio-otic syndrome in South Korea

**DOI:** 10.1038/s41598-023-38909-w

**Published:** 2023-07-21

**Authors:** Somin Lee, Yejin Yun, Ju Hyuen Cha, Jin Hee Han, Dae Hee Lee, Jae-Jin Song, Moo Kyun Park, Jun Ho Lee, Seung Ha Oh, Byung Yoon Choi, Sang-Yeon Lee

**Affiliations:** 1 Department of Otorhinolaryngology-Head and Neck Surgery, Seoul National University Hospital, Seoul National University College of Medicine, Seoul, South Korea; 2 Department of Otorhinolaryngology-Head and Neck Surgery, Seoul National University Bundang Hospital, Seoul, South Korea; 3 CTCELLS, Inc., 21, Yuseong-Daero, 1205 Beon-Gil, Yuseong-Gu, Daejeon, Republic of Korea; 4 Department of Genomic Medicine, Precision Medicine & Rare Disease Center, Seoul National University Hospital, Jongno-Gu, Daehak-Ro, 101, Seoul, South Korea; 5 Sensory Organ Research Institute, Seoul National University Medical Research Center, Seoul, South Korea

**Keywords:** Computational biology and bioinformatics, Genetics, Molecular biology

## Abstract

Branchio-oto-renal (BOR)/branchio-otic (BO) syndrome is a rare disorder and exhibits clinically heterogenous phenotypes, marked by abnormalities in the ear, branchial arch, and renal system. Sporadic cases of atypical BOR/BO syndrome have been recently reported; however, evidence on genotype–phenotype correlations and molecular mechanisms of those cases is lacking. We herein identified five *SIX1* heterozygous variants (c.307dupC:p.Leu103Profs*51, c.373G>A:p.Glu125Lys, c.386_391del:p.Tyr129_Cys130del, c.397_399del:p.Glu133del, and c.501G>C:p.Gln167His), including three novel variants, through whole-exome sequencing in five unrelated Korean families. All eight affected individuals with *SIX1* variants displayed non-syndromic hearing loss (DFNA23) or atypical BO syndrome. The prevalence of major and minor criteria for BOR/BO syndrome was significantly reduced among individuals with *SIX1* variants, compared to 15 BOR/BO syndrome families with *EYA1* variants. All *SIX1* variants interacted with the EYA1 wild-type; their complexes were localized in the nucleus except for the p.Leu103Profs*51 variant. All mutants also showed obvious but varying degrees of reduction in DNA binding affinity, leading to a significant decrease in transcriptional activity. This study presents the first report of *SIX1* variants in South Korea, expanding the genotypic and phenotypic spectrum of *SIX1* variants, characterized by DFNA23 or atypical BO syndrome, and refines the diverse molecular aspects of *SIX1* variants according to the EYA1–SIX1–DNA complex theory.

## Introduction

Branchio-oto-renal (BOR) and branchio-otic (BO) syndrome is a rare disorder that is clinically heterogeneous, characterized by anomalies of the ear, branchial arch, and renal system^1^. In some instances, patients exhibit symptoms that resemble those of BOR syndrome but without renal anomalies; these patients are diagnosed with either branchio-oto syndrome-1^2^ (BOS1; OMIM#602588) or branchio-oto syndrome-3^3^ (BOS3; OMIM#608389). The diagnosis of BOR/BO syndrome can be made based on at least three major criteria or two major and two minor criteria^4^. The major diagnostic criteria include deafness (98.5%), branchial anomalies (49–73%), preauricular pits (53–83%), and renal anomalies (38–70%). The minor criteria include external, middle, and inner ear anomalies and preauricular tags. Some patients present with an atypical form of BOR/BO syndrome, which does not meet the standard diagnostic criteria despite carrying a pathogenic variant of a causative gene related to BOR/BO syndrome. BOR/BO syndrome is characterized by a high penetrance of hearing impairment, with over 90% of individuals affected^4,5^. The type of hearing loss can be classified as mixed (50%), conductive (30%), or sensorineural (20%), and ranges in severity from mild to profound^4,5^.

The genetic landscape of BOR/BO syndrome is complex and varied. Since *EYA1* was identified as the initial BOR/BO syndrome gene^6,7^, more loci have been mapped within the *SIX* gene family, including *SIX1*^8^ and *SIX5*. Although *EYA1* pathogenic variants are the primary cause^6,7^, affecting 40–75% of patients, *SIX1* accounts for 3.0–4.5% of cases^8,9^. Some *SIX5* variants have also been reported, contributing to 0–3.1% of BOR/BO syndrome cases^10,11^.

The SIX1 transcription factor is essential for regulating transcription in the nucleus^12–16^. EYA1 acts as a cofactor and binds to SIX1, forming a bipartite transcription factor^17,18^. The SIX1 protein consists of two conserved domains: the Six domain (SD)^19^, which binds to the EYA1 Eya domain (ED)^20^ for protein–protein interactions, and the DNA-binding homeodomain (HD)^7,8,12,17,18^. The EYA1–SIX1–DNA complex regulates transcription and target genes involved in the development of the branchial arch and otic and renal systems^6,9^.

Despite the significance of *SIX1* variants in the pathogenesis of BOR/BO syndrome, there is scant evidence regarding genotype–phenotype correlation and molecular mechanisms. The phenotypic variability, ranging from non-syndromic hearing loss to typical BOR/BO syndrome, complicates establishing clear correlations. Furthermore, fewer than 10 *SIX1* variants have been functionally characterized according to the EYA1-SIX1-DNA complex theory^8,21,22^. Consequently, additional reports on *SIX1* variants may help delineate the range of *SIX1*-related phenotypes and define the phenotypic characterization associated with non-syndromic hearing loss (DFNA23) or atypical BO syndrome. Moreover, further investigations into *SIX1* variants contribute to a more comprehensive understanding of the underlying molecular genetic mechanisms.

We herein identified five *SIX1* heterozygous variants (c.307dupC:p.Leu103Profs*51, c.373G>A:p.Glu125Lys, c.386_391del:p.Tyr129_Cys130del, c.397_399del:p.Glu133del, and c.501G>C:p.Gln167His), including three novel variants, through whole-exome sequencing in five unrelated Korean families. For comparative analysis, we also included 15 additional Korean BOR/BO syndrome families with *EYA1* variants, revealing the phenotypic characteristics of *SIX1* variants. In addition, we investigated the functional consequences of *SIX1* variants on protein structure, expression, subcellular localization, protein–protein interactions, DNA-binding affinity, and transcriptional activity to elucidate the molecular mechanisms of *SIX1* variants according to the EYA1–SIX1–DNA complex theory.

## Materials and methods

### Study subjects

This study employed a retrospective design utilizing in-house databases of genetic hearing loss from two participating tertiary hospitals. The study was approved by the Institutional Review Boards of Seoul National University Hospital (IRB-H-0905-041-281) and Seoul National University Bundang Hospital (IRB-B-1007-105-402). Written informed consent was obtained from all participants or their legal guardians. All methods were carried out in accordance with relevant guidelines and regulations. Molecular genetic testing of 1280 probands was conducted, independent of audiology phenotypes and inheritance patterns^23^. We focused on probands with pathogenic *SIX1* variants. Consequently, we identified five unrelated Korean families (approximately 50%) with segregating as a dominant trait. To further elaborate the clinical phenotypes of *SIX1* variants, our study included 15 more Korean families (encompassing 16 affected individuals) who possessed causative *EYA1* variants linked to BOR/BO syndrome from the in-house database^24^. These additions enabled a comparative analysis of the phenotypic manifestations between individuals with *SIX1* and *EYA1* variants.

### Molecular genetic testing

Genomic DNA was extracted from peripheral blood using a standard procedure, and then subjected to whole-exome sequencing. The reads were aligned using the University of California Santa Cruz hg19 reference genome browser (https://genome.ucsc.edu/) with Lasergene ver. 14 software (DNASTAR, Madison, WI, USA). As described previously^25–27^, strict filtering and bioinformatics were performed to retrieve genetic etiologies. Candidate variants were validated using Sanger sequencing, and segregation studies were performed using paternal DNA samples when possible. All variants identified were classified in accordance with the ACMG/AMP guidelines for hearing loss^28,29^.

### Structural modeling

The model structure of the EYA1-SIX1 complex was generated using the Protein Structure Database^30,31^. The interaction between EYA1 and SIX1 was analyzed by aligning the EYA1 model structure to the EYA2–SIX1 complex structure (4EGC)^32^. To analyze the changes of *SIX1* variant in DNA binding interface, homeodomain of SIX1 model structure is superimposed with Exd homeodomain from AbdB/Exd-DNA complex structure (5ZJQ)^33^. The residues corresponding to the unidentified linker regions (p.Lys114-p.Phe131) were not available for generating a 3D structure model. Thus, among the identified variants, the impact of the novel *SIX1* missense variant (p.Gln167His) on stability was predicted by comparing intramolecular interactions, such as hydrogen bonding and cation-π interactions, using the PyMOL software (v. 2.4.1; PyMOL Molecular Graphics System v. 2.0, Schrödinger Inc., New York, NY, USA).

### Plasmids, cell culture, and transfection

A human SIX1 cDNA clone (RC203465) and an EYA1 Human Tagged ORF Clone (RC219782) was purchased from Origene. The *SIX1* variant plasmids, including pCMV6-Myc-DDK entry, pCMV6-SIX1 wild-type-Myc-DDK, pCMV6-SIX1 p.Leu103Profs*51-Myc-DDK, pCMV6-SIX1 p.Glu125Lys-Myc-DDK, pCMV6-SIX1 p.Tyr129_Cys130del-Myc-DDK, pCMV6-SIX1 p.Glu133del-Myc-DDK, and pCMV6-SIX1 p.Gln167His-Myc-DDK, were generated utilizing the QuickChange mutagenesis method^34^. Furthermore, the EYA1 plasmid was subcloned to include a 6xHis tag at the C-terminal, in place of the Myc-DDK tag. HEK293 cells were cultured in DMEM (LM001-05, Celgene) supplemented with 10% fetal bovine serum (12483-020, Gibco), 100 units/mL penicillin/streptomycin (LS015-01, Welgene), and 2 mM l-glutamine (LS002-01, Welgene). The cells were maintained in a humidified atmosphere containing 5% CO_2_ at 37 °C. For transient overexpression, cells were transfected with 0.5–1 µg of total plasmid DNA in a 12-well culture plate (> 95% confluent or at a density of 106 cells/well) for 24 h using jetPRIME reagent (101000015, Polyplus), in accordance with the manufacturer's guidelines.

### Immunocytochemistry

For immunofluorescence microscopy, cultured HEK293 cells on a cover glass were transfected with 1–2 μg of total plasmid DNA for 24 h. After that, the cells were fixed with 4% paraformaldehyde for 20 min, permeabilized with 0.5% Triton X-100 in PBS for 20 min, and blocked with 1% BSA in PBS for 20 min. And then the cells were incubated with the primary antibodies for overnight. Following incubation, the cells were washed 3-times for 10 min with PBS and incubated with Alexa Fluor-conjugated secondary antibody diluted in 1% BSA/PBS for 40 min at room temperature. The cells were then mounted with 4′,6′-diamidino-2-phenylindole (DAPI)-containing mounting medium (ab104139, abcam). Confocal images were captured by a laser scanning confocal microscope (Leica STELLARIS 8, Upright).

### Western blotting

Proteins in whole cell lysates were separated by 7–13% sodium dodecyl sulfate–polyacrylamide gel electrophoresis (SDS-PAGE) and transferred to 0.45 μm polyvinylidene difluoride (PVDF) membranes (IPVH00005, Millipore). The membranes were incubated with 5% skim-milk at room temperature for 1 h and probed with the following primary antibodies: anti-Myc (2276S, CST), anti-His (12698S, CST), anti-β-actin (sc-47778, Santa Cruz biotechnology). The membranes were incubated with a horseradish peroxidase conjugated anti-mouse IgG antibody (SA001-500, GenDEPOT) or anti-rabbit IgG antibody (SA002-500, GenDEPOT). The protein band were detected by chemiluminescence reagent (RPN2106, cytiva). The band intensity was measured by Image J software.

### His-tagged protein pull-down assay

HEK293 cells were transfected with 1 μg his-tagged EYA1 plasmids and with 1 μg of plasmids expressing Myc-flag-tagged *SIX1* variants. Whole cell lysates were prepared in the lysis buffer (25 mM Tris–HCl pH 7.4, 150 mM NaCl, 1% NP-40 and 5% glycerol) and then mixed with TALON Metal Affinity Resin (635501, Clontech) for 3 h. The co-precipitated proteins were examined by SDS-PAGE and immunoblotting after the beads were washed three times with the same buffer. Following three washes of the beads with the identical buffer, the co-precipitated proteins were subjected to analysis using SDS-PAGE and immunoblotting.

### DNA binding assay

The Abcam nuclear extraction kit (ab113474) was used, following the manufacturer's protocol, to obtain nuclear extracts from the cells. Subsequently, these nuclear extracts were employed in a DNA binding assay using the DNA–Protein Binding Assay Kit (Colorimetric) (ab117139), outlined by the provided protocol, with the aim of assessing the binding affinity of the SIX1 mutant proteins to DNA. In this experiment, 8.5 ug of nuclear extracts and 30 ng of biotin-labeled double stranded DNA were used for binding assay. Fold changes were normalized by using the raw values of nuclear extracts obtained from non-transfected control cells as a control.

### Luciferase reporter assay

As previously described^35,36^, the pGL4.12[luc2CP]-MYOG-6xMEF3 construct, which comprises a luciferase reporter and six repeats of the MEF3 motif that binds to the SIX1 protein in a cell culture system, was utilized in this experiment. The luciferase assay was carried out in accordance with the protocol provided by the manufacturer (E1500, Promega). In brief, HEK293T cells transfected with pGL4.12[luc2CP]-MYOG-6xMEF3, pCMV6-SIX1 mutant constructs, and pCMV6-EYA1 wild-type were lysed using 1 × lysis reagent. Subsequently, 20 µl of cell lysate was mixed with 100 µl of Luciferase Assay Reagent, and the resulting light production was measured using the Glomax 20/20 Luminometer (Promega). The luciferase activity, indicative of transcriptional activity, of the wild-type and each SIX1 mutant was normalized to an internal control consisting of Myc-DDK-transfected empty vector.

### Ethics approval

The study was approved by the Institutional Review Boards of Seoul National University Hospital (IRB-H-0905-041-281) and Seoul National University Bundang Hospital (IRB-B-1007-105-402).

## Results

### Clinical phenotypes

**Figure 1:**
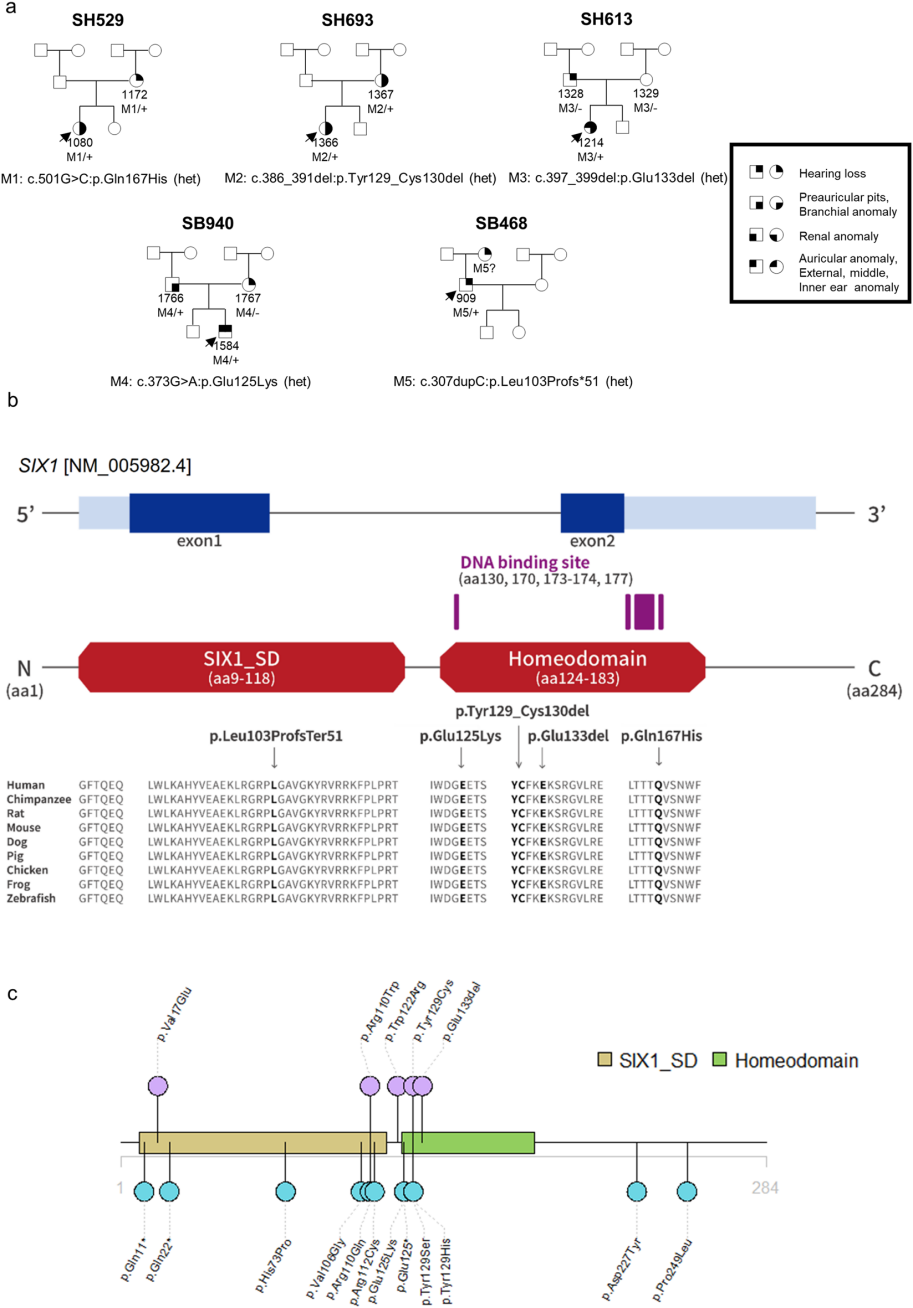
Clinical phenotypes of the five unrelated Korean families segregated with *SIX1* variants and functional characterization of all the *SIX1* variants. (**a**) The pedigrees of the five unrelated Korean families with *SIX1* variants. The clinical phenotypes were summarized based on major and minor diagnostic criteria for BOR/BO syndrome. (**b**) Protein domain and conservation maps. The residues of the five *SIX1* variants were in the SIX1 SD or HD domains. One truncating variant (p.Leu103Profs*51) was in the SIX1 SD domain. All the variants’ residues were well-conserved among the SIX1 orthologs in various species. (**c**) *SIX1* variants reported thus far in the literature. The upper color (purple) represents the *SIX1* variants that have been studied functionally, while the lower color (blue) denotes *SIX1* variants that have not yet been characterized functionally. This study presents five variants. Among them, three variants (p.Leu103Profs*51, p.Tyr129_Cys130del, and p.Gln167His) are novel, and two variants (p.Glu125Lys and p.Glu133del) have previously been reported. The Glu133del variant has undergone functional studies.

This study included eight affected patients (three males and five females) from five unrelated families, segregating with *SIX1* variants (Fig. 1a). A comprehensive overview of their clinical phenotypes, encompassing both major and minor criteria, is provided in Table 1. All eight individuals (100%) experienced sensorineural hearing loss (SNHL), with three (37.5%) having preauricular pits and one (12.5%) presenting with branchial anomalies. Kidney imaging was performed on all patients, revealing no significant findings such as hypoplasia or multi-cystic dysplastic kidneys. Following physical examinations and temporal bone computed tomography (CT) scans, only two patients (25%) showed inner ear anomalies, which included incomplete cochlear turn, cochlear hypoplasia, and enlarged vestibular aqueduct (EVA). In contrast, the patients had no external or middle ear anomalies. All patients exhibited an atypical BOR/BO syndrome, indicating a milder phenotypic spectrum. Specifically, 50% of the affected patients displayed DFNA23, aligning with either one major or one major and one minor criterion.

**Table 1:** Detailed clinical features of *SIX1* and *EYA1* variants according to criteria BOR/BO syndrome.

Family no.	Sex/age	Gene	Variant	Branchial anomalies	Preauricular pits	Hearing loss (type)	Renal anomalies	External	Middle	Inner	EVA	Typical vs. atypical^a^
SH529-1080	F/31	SIX1	c.501G>C:p.Gln167His	O	–	SNHL	–	–	–	–	–	Atypical
SH529-1172	F/60	SIX1	c.501G>C:p.Gln167His	–	–	SNHL	–	–	N/A	N/A	N/A	Atypical
SH693-1366	F/10	SIX1	c.386_391del:p.Tyr129_Cys130del	–	O	SNHL	–	–	–	N/A	N/A	Atypical
SH693-1367	F/34	SIX1	c.386_391del:p.Tyr129_Cys130del	–	O	SNHL	–	–	–	N/A	N/A	Atypical
SH613-1214	F/11	SIX1	c.397_399del:p.Glu133del	–	O	SNHL	–	–	–	O	–	Atypical
SB940-1584	M/32m	SIX1	c.373G>A:p.Glu125Lys	–	–	SNHL	–	–	–	O	O	Atypical
SB940-1766	M/28	SIX1	c.373G>A:p.Glu125Lys	–	–	SNHL	–	–	–	N/A	N/A	Atypical
SB468-909	M/66	SIX1	c.307dupC:p.Leu103ProfsTer51	–	–	SNHL	–	–	–	N/A	N/A	Atypical
SH527-1078	F/16	EYA1	c.1319G>A;p.Arg440Gln	O	O	MHL	O	–	N/A	O	–	Typical
SH114-234	M/17	EYA1	c.1220G>A:p.Arg407Gln	O	O	MHL	O	O	O	O	–	Typical
SH751-1487	F/9	EYA1	c.1081C>T:p.Arg361Ter	O	O	MHL	–	O	O	O	O	Typical
SH751-1488	M/9	EYA1	c.1081C>T:p.Arg361Ter	O	O	MHL	–	–	O	O	–	Typical
SH751-1489	M/6	EYA1	c.1276G>A:p.Gly426Ser	–	–	N/A	–	O	–	–	–	Atypical
SH751-1490	M/9	EYA1	c.1276G>A:p.Gly426Ser	O	–	–	N/A	–	N/A	N/A	N/A	Atypical
SH215-499	F/12	EYA1	Deletion	O	O	MHL	–	O	O	O	–	Typical
SH468-989	M/8	EYA1	c.1081C>T:p.Arg361Ter	–	O	MHL	O	O	O	O	–	Typical
SH536-1087	F/23	EYA1	Inversion reciprocal with deletion	O	O	MHL	N/A	–	O	O	O	Typical
SH536-1091	F/51	EYA1	Inversion reciprocal with deletion	–	O	N/A	–	O	N/A	N/A	N/A	Atypical
SH536-1094	F/29	EYA1	Inversion reciprocal with deletion	O	O	N/A	N/A	–	N/A	O	O	Typical
SH536-1093	M/32	EYA1	Inversion reciprocal with deletion	O	O	SNHL	O	–	N/A	O	O	Typical
SH587-1179	M/32	EYA1	c.1623_1626dup:p.Gln543AsnfsTer90	O	O	SNHL	–	–	–	O	–	Typical
SB516-982	M/21	EYA1	c.1360G>T:p.Gly454Cys	–	–	SNHL	O	O	–	O	O	Typical
SB651-1164	F/6	EYA1	c.1117_1118delCA:p.His373PhefsTer4	–	O	MHL	–	O	O	O	O	Typical

*SIX1* canonical transcript NM_005982.4, *EYA1* canonical transcript NM_000503.6.

*F* female, *M* male, *SNHL* sensorineural hearing loss, *MHL* mixed hearing loss, *EVA* enlarged vestibular aqueducts, *N/A* not available.

^a^Note, if the note doesn't satisfy the standard criteria for BOR/BO syndrome (at least three major criteria or two major and two minor criteria), it can be classified as atypical BOR/BO syndrome.

**Figure 2:**
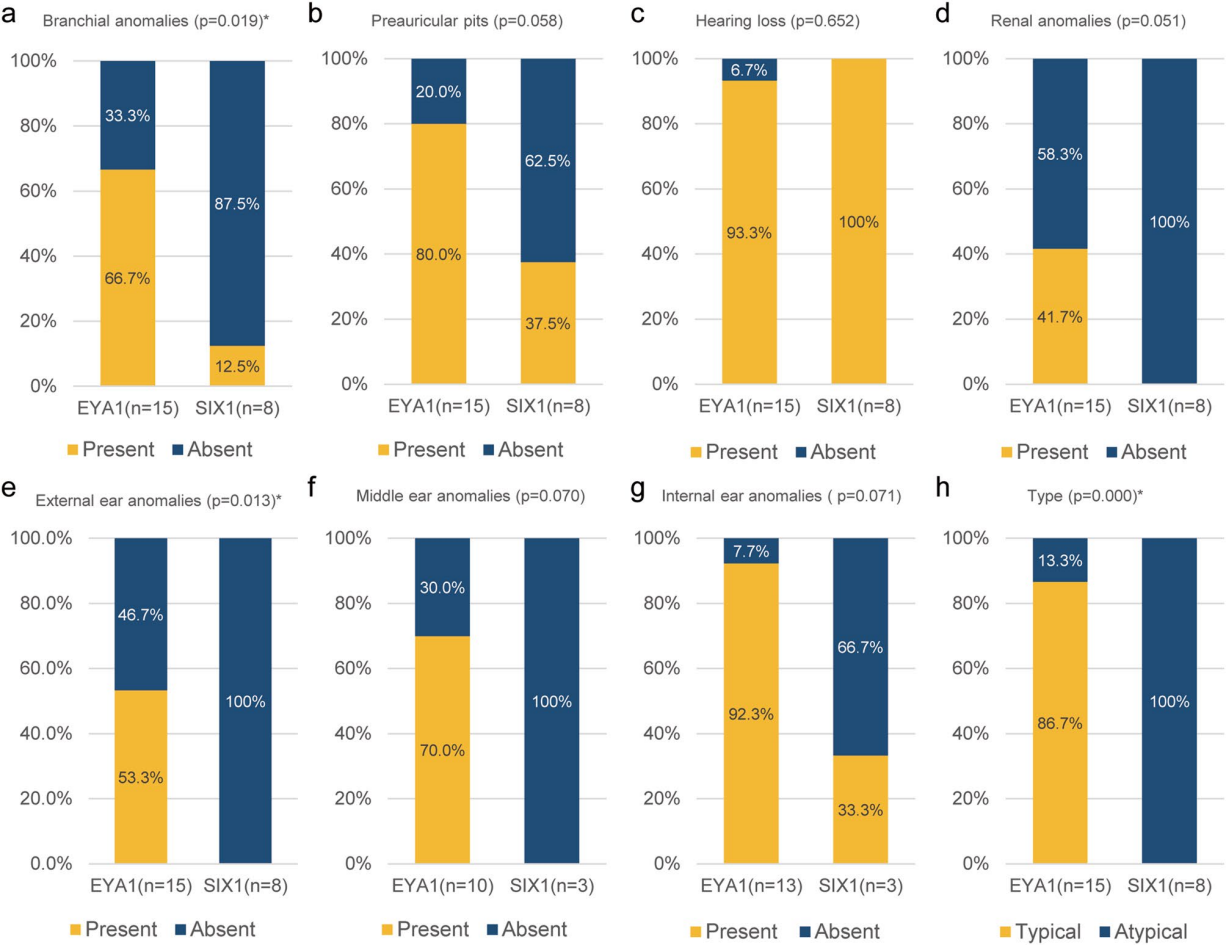
Comparison of clinical profiles between the *SIX1* and *EYA1* variants using an in-house database (**a**–**d**) A comparative analysis of the prevalence of major diagnostic criteria for BOR syndrome among individuals with *SIX1* and *EYA1* variants. (**e**, **f**) A comparison evaluating the prevalence of minor diagnostic criteria for BOR syndrome between individuals with *SIX1* and *EYA1* variants. (**h**) Phenotypic features associated with *SIX1* variants in relation to DFNA23 or atypical BO syndrome.

We compared the clinical profiles of cohorts with *EYA1* variants (15 affected patients from 10 unrelated families) using our in-house database to further clarify the link between *SIX1* variants and milder phenotypes. Qualitative analysis showed that the incidence of branchial and external ear anomalies was significantly reduced in the *SIX1* group compared to the *EYA1* group (p = 0.019 and p = 0.013, respectively) (Fig. 2a,b,e). Moreover, the prevalence of preauricular pits, renal anomalies, and middle ear and inner ear anomalies was also noticeably lower in the *SIX1* group than in the *EYA1* group (p = 0.058, p = 0.051, p = 0.070, p = 0.071, respectively) (Fig. 2b,d,f,g), despite lack of statistically significance. No significant difference was observed in cofactors such as age at ascertainment or sex between the two groups. The lower prevalence of major and minor criteria, excluding one major criterion (i.e., hearing loss) (Fig. 2c), in patients with *SIX1* variants supports the link between these *SIX1* variants and a milder phenotype of BOR/BO syndrome. This finding provides clinical insights aiding identification of *SIX* variants in individuals without typical BOR/BO syndrome. Our findings shed further light on the genotype–phenotype correlation in DFNA23 or atypical BO syndrome caused by *SIX1* variants (Fig. 2h).

We further evaluated the exhaustive account of the *SIX1*-related phenotypes reported in the literature in Table S1. We also provided a comparison of statistics for each phenotype in this study versus those in other studies in Fig. S1. Notably, no significant difference in the overall phenotypes was observed. These data serve to improve the clarity and consistency of our results, highlighting that *SIX1* variants were significantly associated with DFNA23 or atypical BO syndrome.

### Genotypes

We identified five SIX1 heterozygous mutant alleles (p.Leu103Profs*51, p.Glu125Lys, p.Tyr129_Cys130del, p.Glu133del, and p.Gln167His) through whole-exome sequencing (Fig. 1a). Three were novel variants, including a frameshift variant (p.Leu103Profs*51), an inframe deletion (p.Tyr129_Cys130del), and a missense variant (p.Gln167His). The other mutant alleles (p.Glu125Lys and p.Glu133del) had previously been reported as pathogenic or likely pathogenic based on the ACMG/AMP guidelines^28,29^. All *SIX1* variants were in regions encoding the SD or HD domains, with one (p.Leu103Profs*51) being located in the SD domain and four (p.Glu125Lys, p.Tyr129_Cys130del, p.Glu133del, and p.Gln167His) in the HD domain. Presumably, the SD α6 and HD domains and the linker region connecting them are hotspot regions that cause many mutants. Additionally, all variants were well-conserved among the SIX1 orthologs in various species (Fig. 1b). Seventeen SIX1 variants have been reported (Fig. 1c); functional studies investigated the five variants (Fig. 1c). The novel variants were extremely rare in population databases, including ethnically matched databases. In silico prediction tools and conservation analysis predicted that these variants were likely to have a detrimental effect on protein structure and function. The frameshift variant (p.Leu103Profs*51) truncated the SD α6 and HD domains and was predicted to undergo nonsense-mediated mRNA decay in vivo. Three novel variants, including p.Leu103Profs*51, p.Tyr129_Cys130del, and p.Gln167His, were considered “pathogenic” or “likely pathogenic” based on the ACMG/AMP guidelines (Table 2).

**Table 2:** *SIX1* variants in the current study and in-silico prediction analysis.

Proband	Genomic position: change (GRCh37/hg19)	HGVS		Location (exon/domain)	Zygosity/inheritance	In-silico predictions		Allele frequency			ACMG/AMP 2018 guideline	
		Nucleotide change	Amino acid change			CADD Phred	REVEL	KRGDB	KOVA	gnomAD	Criteria	Classification
SH529-1080	Chr14:61115407C>G	c.501G>C	p.Gln167His	Exon1/homeodonain (HD)	Het/autosomal dominant	28.5	0.919	Absent	Absent	Absent	PS3, PM2, PP3	Likely pathogenic
SH693-1366	Chr14:61115517 AGCAGT	c.386_391del	p.Tyr129_Cys130del	Exon1/homeodonain (HD)	Het/autosomal dominant	NA	NA	Absent	Absent	Absent	PS3, PM2, PP4	Likely pathogenic
SB468-909	Chr14:61115601	c.307dupC	p.Leu103ProfsTer51	Exon1/six domain (SD)	Het/autosomal dominant	NA	NA	Absent	Absent	Absent	PVS1, PS3, PM2, PP4	Pathogenic
SB940-1584	Chr14:61115535	c.373G>A	p.Glu125Lys	Exon1/homeodonain (HD)	Het/autosomal dominant	26.0	0.794	Absent	Absent	Absent	PS3, PM2, PP3	Likely pathogenic
SH613-1214	Chr14:61115508TCTC>T	c.397_399del	p.Glu133del	Exon1/homeodonain (HD)	Het/autosomal dominant	NA	NA	Absent	Absent	Absent	PS2_supporting, PS3, PM2	Likely pathogenic

*Het* heterozygote, *VUS* variant uncertain significance, *NA* not available.

Refseq transcript accession number NM_005982.4; Refseq protein accession number NP_005973.

*HGVS* Human Genome Variation Society (https://www.hgvs.org/).

Sequence Variant Nomenclature (https://mutalyzer.nl/).

*CADD* Combined Annotation Dependent Depletion (https://cadd.gs.washington.edu/).

*REVEL* Rare Exome Variant Ensemble Learner (https://sites.google.com/site/revelgenomics/).

*KRGDB* Korean Reference Genome Database (http://152.99.75.168:9090/KRGDB/welcome.jsp).

*KOVA* Korean Variant Archive for a reference database of genetic variations in the Korean population (https://www.kobic.re.kr/kova/).

*gnomAD* The Genome Aggregation Database (https://gnomad.broadinstitute.org/).

### Structure basis of *SIX1* variants

**Figure 3:**
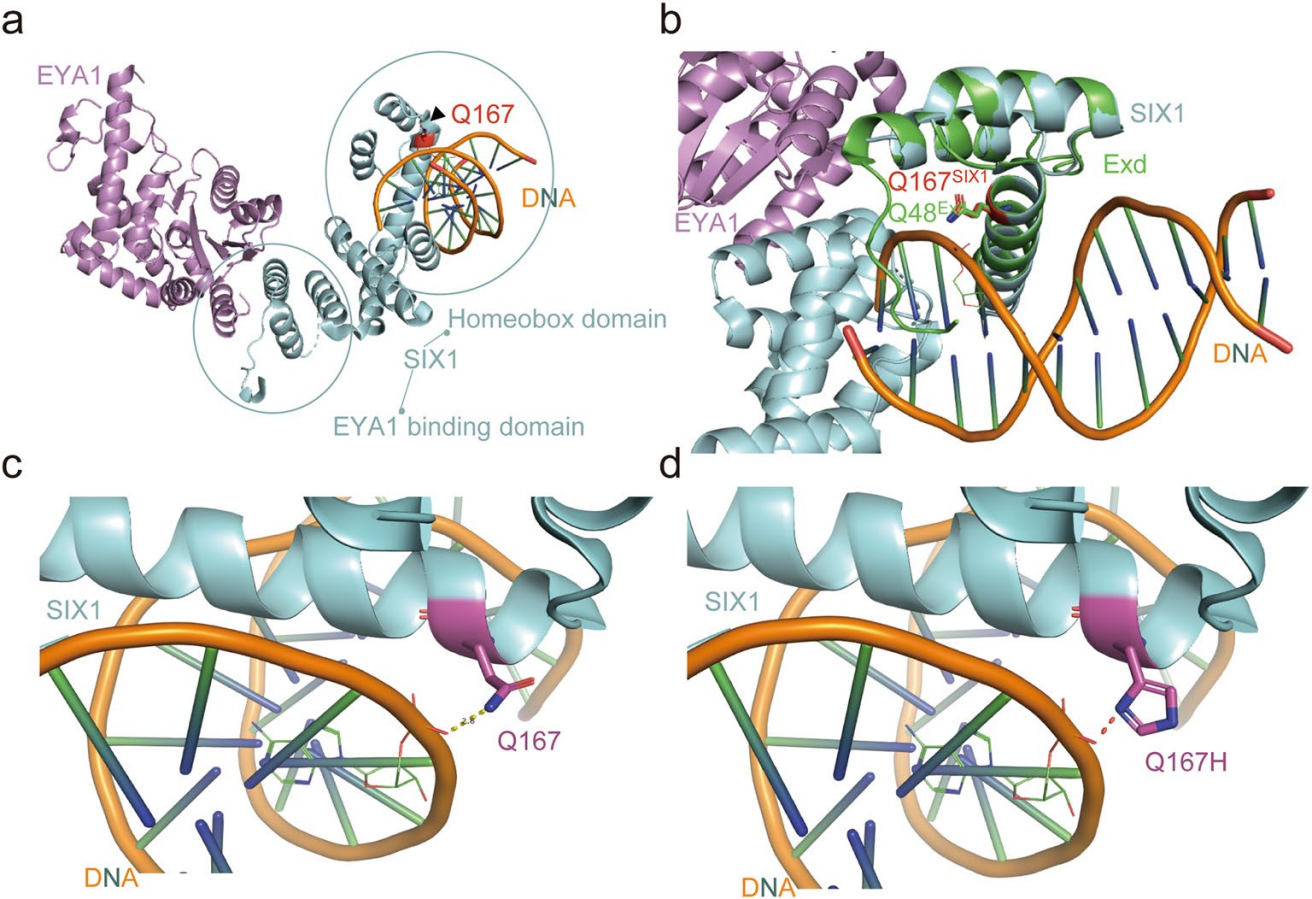
Three-dimensional modeling and structural analysis. All four SIX1 mutants in the homeodomain produce structural changes that sterically inhibit DNA binding, possibly compromising structure stability. (**a**) SIX1 is important for the overall DNA interaction processes of the HD domain. (**b**) Modeling by superimposing the SIX1-DNA complex over the AbdB/Exd-DNA complex revealed close alignment with the SIX1 HD domain. (**c**) The hydrogen bonds between the DNA phospho-backbone and p.Gln167 stabilize the DNA duplex. (**d**) The bulky side chain of the p.Gln167His variant expels itself from the DNA binding cleft, leading to a loss of hydrogen bonds.

The pathogenicity of causative *SIX1* variants underlying BOR/BO syndrome, previously reported to map to either the HD domain or the α6 in the SD domain, is well documented^32^. We evaluated the conservation of the p.Gln167 residue to investigate the pathogenicity of the *SIX1* p.Gln167His variant. *SIX1* p.Gln167 is highly conserved among more than 120 human transcription factor HDs in its corresponding residue in the helix C^37^. This indicates that *SIX1* p.Gln167 plays a crucial role in the overall DNA interaction processes of the HD domain (Fig. 3a). To understand the structural implications of the *SIX1* p.Gln167His variant for DNA interactions, we modeled the SIX1–DNA complex structure by superimposing it on the AbdB/Exd–DNA complex. Exd HD, a well-known example of a homeobox protein, aligned well with the SIX1 HD domain (Fig. 3b). The hydrogen bonds between p.Gln167 and the DNA phospho-backbone maintained stability in the DNA duplex (Fig. 3c). In contrast, the bulky side chain of the p.Gln167His variant expelled itself from the DNA binding cleft, leading to a loss of hydrogen bonds between SIX1 and the DNA phosphor backbone (Fig. 3d). Investigation of the SIX1-EYA1 complex structure suggests that the p.Gln167His variant could compromise protein stability and disrupt DNA binding ability.

### Protein expression

**Figure 4:**
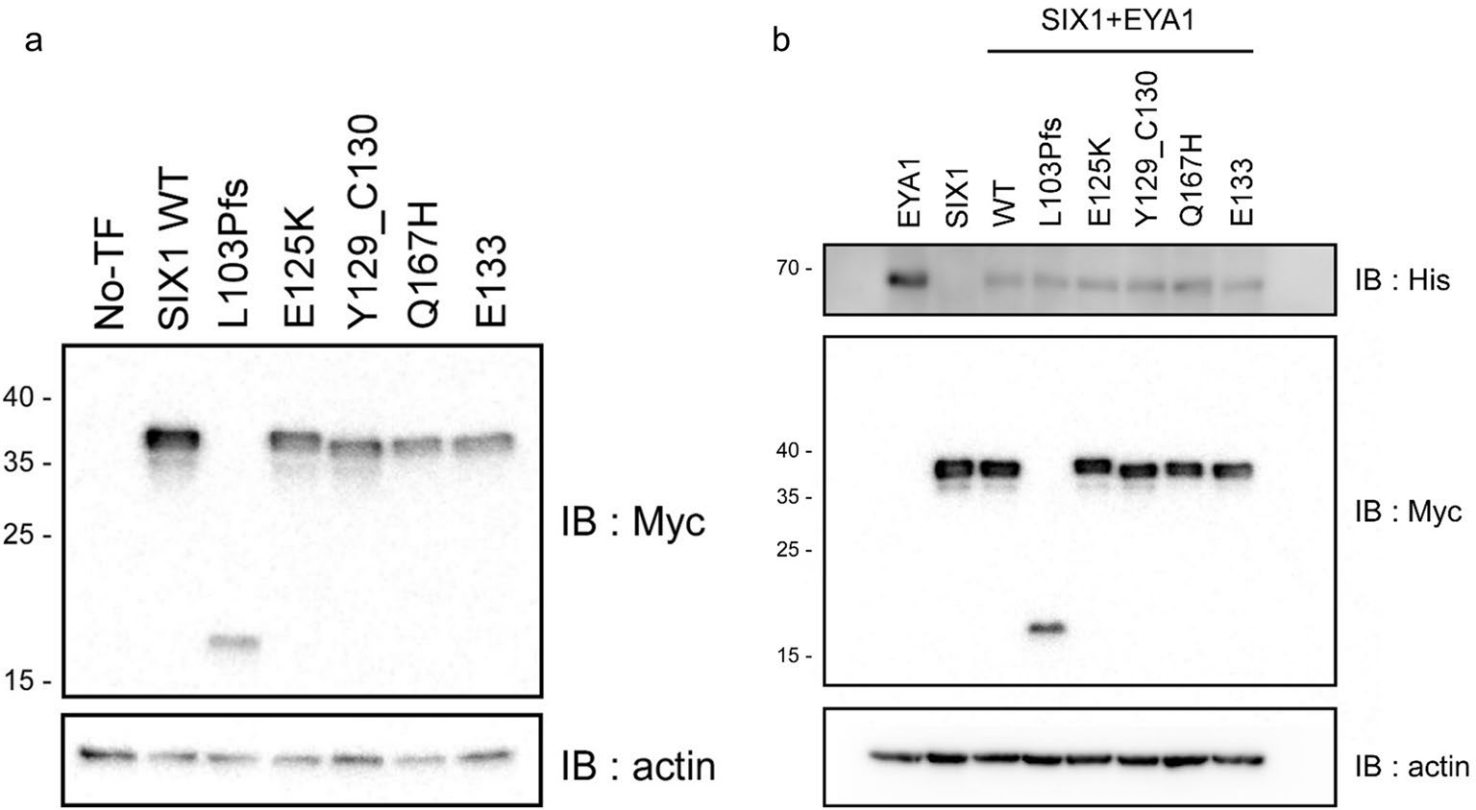
Western blot analysis for the SIX1 wild-type, frameshift, and missense variants by transient transfection of HEK293 cells. (**a**) Expression of SIX1 wild-type and mutants was detected by western blotting of HEK293 cells. The SIX1 wild-type and missense variants have a molecular weight of 42 kDa. The molecular weight of the frameshift variant is 21 kDa. (**b**) Expression of SIX1 wild-type and mutants co-transfected with EYA1 wild-type was detected by western blotting in HEK293 cells. The SIX1 wild-type and missense variants have a molecular weight of 42 kDa, and that of the frameshift variant is 21 kDa. The original immunoblots (uncropped, full length membranes with membrane edges visible, and standard protein size markers and expected molecular weight labeled) were all provided in Fig. S5.

To confirm whether *SIX1* variants are stably expressed in cells, we transfected mammalian cells with plasmids encoding each *SIX1* variant (Fig. 4a). *SIX1* variants were overexpressed in HEK293 cells, and the same amount of plasmid was used for transfection. The level of protein expression was evaluated using sampled whole-cell lysates. Without transient overexpression of EYA1 (i.e., single-transfected state), the expression level of all SIX1 mutants was significantly lower than that of SIX1 wild-type, suggesting that the mutants are more susceptible to degradation and instability. Next, considering that SIX1 functions in conjunction with EYA1 (SIX1 co-factor) to form a bipartite complex^38,39^, we performed immunoblotting to compare the levels of SIX1 protein expression when EYA1 was overexpressed (Fig. 4b). As a result, when EYA1 and SIX1 were co-expressed, it was confirmed that the expression levels of all SIX1 mutants, except for SIX1 p.Leu103Profs*51, were comparable to wild-type protein. This, in turn, suggests that *SIX1* variants can form a stable EYA1-SIX1 complex when EYA1 was overexpressed. On the other hand the expression level of SIX1 p.Leu103Profs*51 was reduced even in the presence of overexpressed EYA1. This finding calls into the question that the *SIX1* truncated variant (p.Leu103Profs*51) could either interfere with the formation of the EYA1-SIX1 complex and/or fail to translocate into the nucleus, which subsequently structural instability prone to degradation.

### Subcellular localization

**Figure 5:**
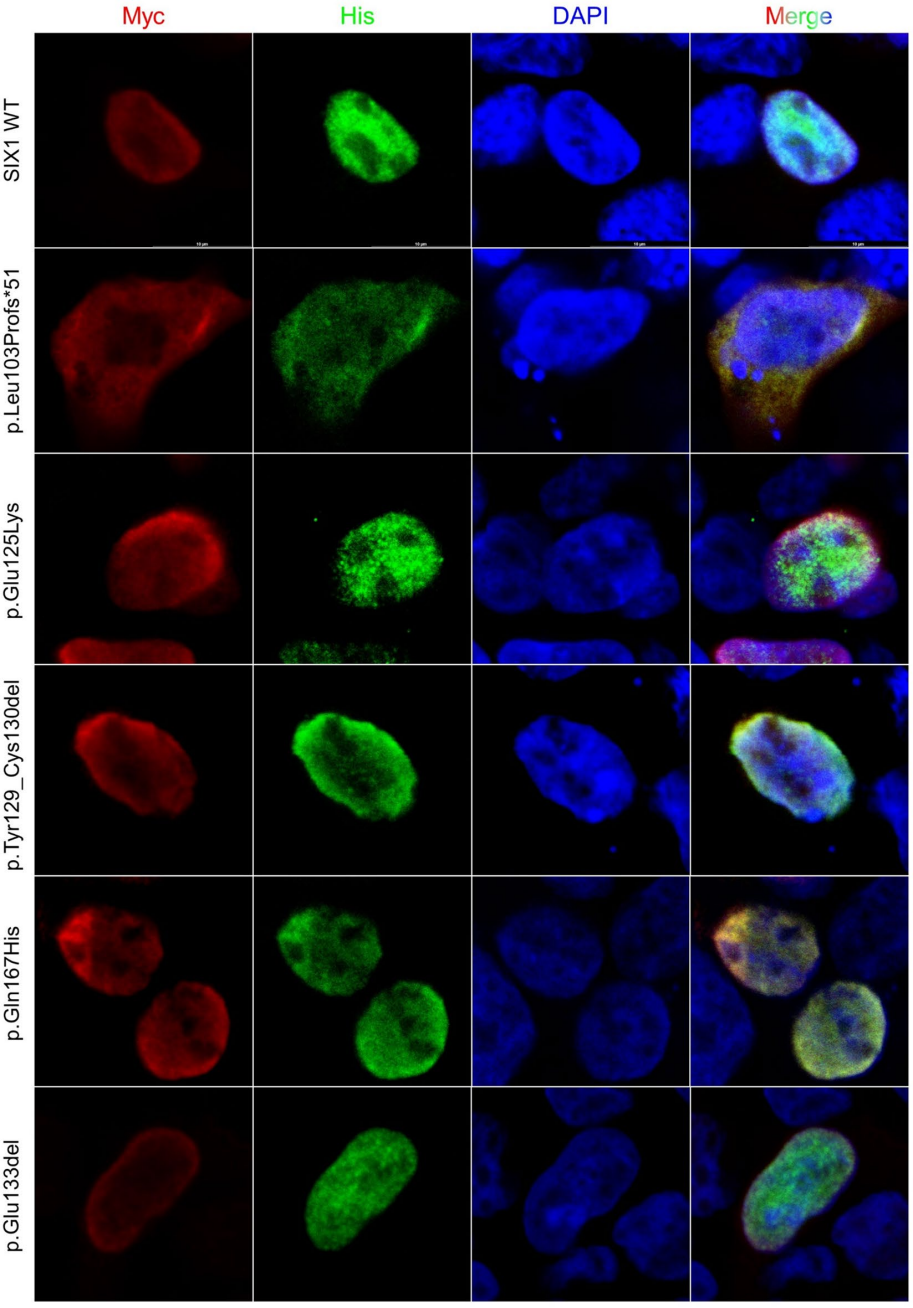
Subcellular localization of SIX1 wild-type and mutants. Immunofluorescence of HEK293 cells co-transfected with C-terminally Myc-DDK-tagged SIX1 wild-type, p.Leu103Pfs*51, p.Glu125Lys, p.Tyr129_Cys130del, p.Gln167His, p.Glu133del, and C-terminally 6xHis-tagged EYA1 wild-type. Cells were immunostained with anti-Myc^30^ and anti-His (green) antibodies. In co-transfections with EYA1 wild-type, all SIX1 mutants and wild-type were localized in the nucleus, except for p.Leu103Profs*51.

This study revealed that in the absence of SIX1, full-length EYA1 localized primarily in the cytoplasm (Fig. S2); however, co-expression with the SIX1 wild-type resulted in its predominantly nuclear localization, consistent with previous reports^17,21^. All SIX1 mutants, except p.Leu103Profs*51, could localize in the nucleus, whether expressed alone or in conjunction with EYA1 wild-type (Fig. 5). The *SIX1* p.Leu103Profs*51 mutant, which affects the SD α6 and HD domains, failed to translocate to the nucleus even in the presence of EYA1 wild-type. This suggests that the disruption of the EYA1-SIX1 complex or the absence of the HD domain may be responsible for the loss of nuclear translocation capacity in the *SIX1* p.Leu103Profs*51 mutant.

### Protein–protein interactions

**Figure 6:**
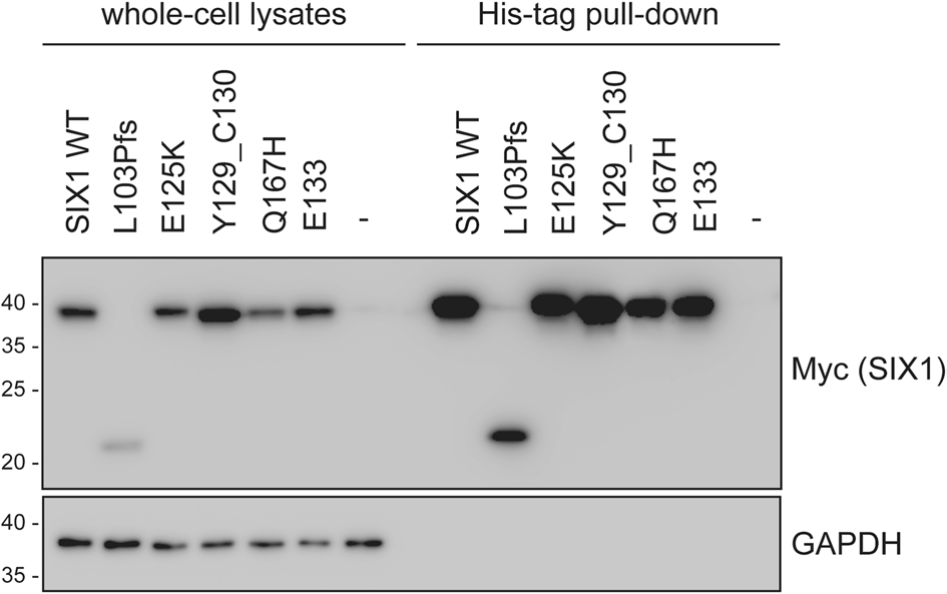
Protein–protein interactions between *EYA1* and *SIX1* variants. HEK293 cells were transfected with His-EYA1 and Myc-DDK-SIX1 plasmids for 24 h. Whole-cell lysates were collected and subjected to protein–protein interaction assays with TALON resin to His-tag protein pull-down. The original immunoblots (uncropped, full length membranes with membrane edges visible, and standard protein size markers and expected molecular weight labeled) were all provided in Fig. S6.

Previous studies verified the interaction between SIX1 and EYA1 by co-immunoprecipitation, gel mobility assays, and immunohistochemistry^40,41^. Considering the importance of the SIX1–EYA1 interaction for proper SIX1 function, we conducted a protein–protein interaction assay to determine whether SIX1 mutants and wild-type EYA1 co-localize in the cell nucleus and interact. We conducted an in vitro pull-down assay to assess the direct interaction between these proteins (Fig. 6). HEK293 cells were transfected with EYA1-His and SIX1-Myc, and whole-cell lysates were prepared. These lysates were incubated with TALON resin to pull down the EYA1-His protein. All *SIX1* variants exhibited substantial interaction with EYA1, including p.Leu103Profs*51, which failed to localize to the nucleus. The SIX1 mutants, where the SD domain was well-conserved, maintained functional interactions with wild-type EYA1, preventing the loss of the SIX1–-EYA1 complex.

### DNA binding affinity

**Figure 7:**
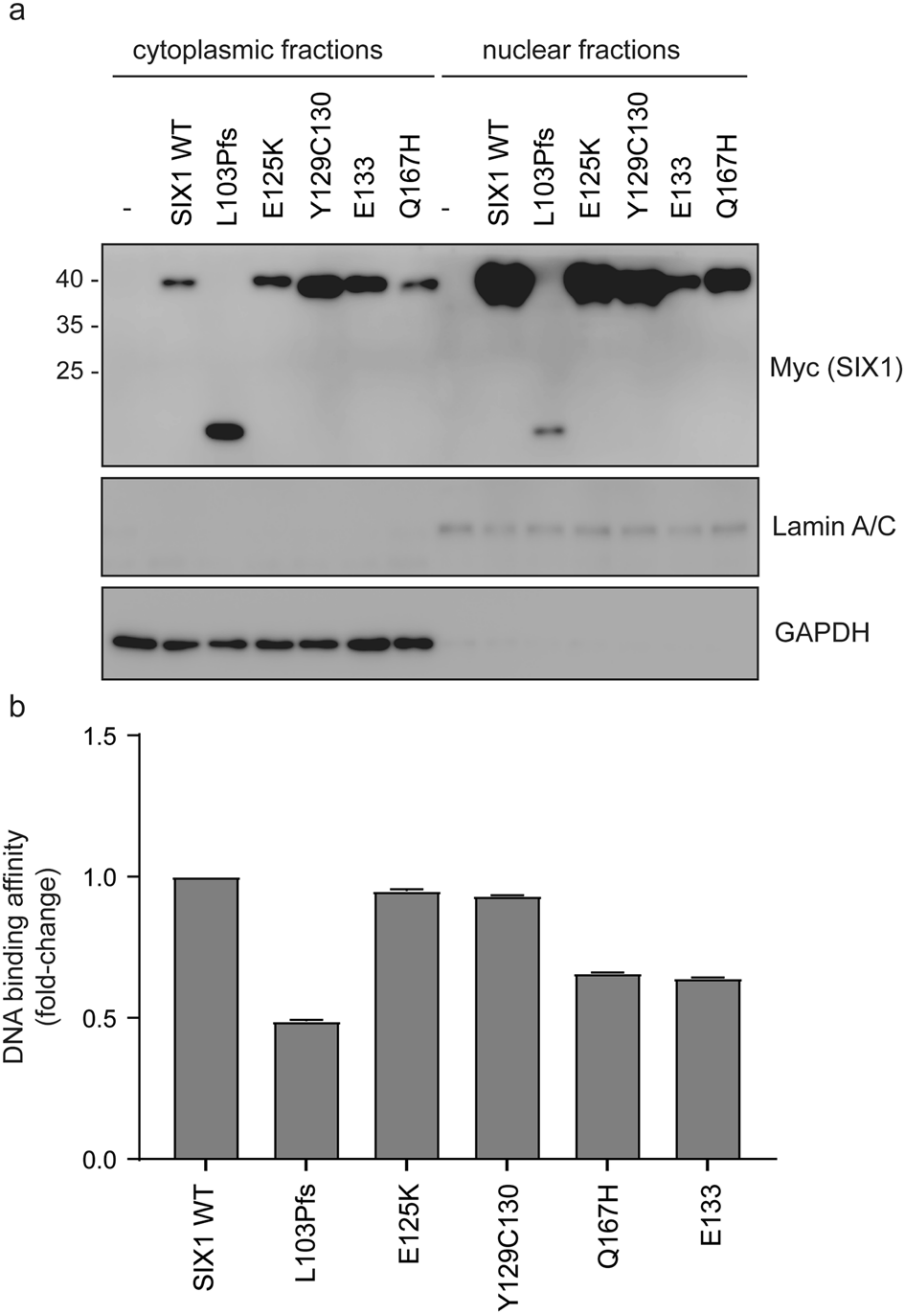
DNA binding assay for SIX1 wild-type variants to measure the binding affinity of SIX1 protein to DNA. (**a**) HEK293 cells were transfected with the *SIX1* variant plasmids, pCMV6-SIX1 wild-type-Myc-DDK, pCMV6-SIX1 p.Leu103Profs*51-Myc-DDK, pCMV6-SIX1 p.Glu125Lys-Myc-DDK, pCMV6-SIX1 p.Tyr129_Cys130del Myc-DDK, pCMV6-SIX1 p.Glu133del-Myc-DDK, and pCMV6-SIX1 p.Gln167His-Myc-DDK, and nuclear extracts were obtained. The original immunoblots (uncropped, full length membranes with membrane edges visible, and standard protein size markers and expected molecular weight labeled) were all provided in Fig. S7. (**b**) Anti-Myc antibody (2276S, CST) was incubated with nuclear extracts to quantify the binding affinity of SIX1 protein to DNA colorimetrically. Nuclear extracts of non-transfected cells were used as a control. The experiment was conducted in triplicate, and data were analyzed by one-way ANOVA.

Next, we tested the DNA binding affinities of SIX1 variants required for transcriptional activityo determine the characteristics of SIX1 in its function as a transcription factor. After overexpression of EYA1 wild-type and SIX1 mutants on HEK293 cells, we fractionated whole-cell lysates into cytoplasmic and nuclear fractions. The successfulness of the fractionation process was validated by evaluating the levels of GAPDH, an indicator of the cytoplasm indicative of the cytoplasm, and Lamin A/C, a marker for the nucleus (Fig. 7a). Remarkably, except for the SIX1 p.Leu103Profs*51 variant which failed to nuclear localization, the presence of EYA1 protein, all variants were confirmed to be stably localized in the nucleus when co-expressed fraction stably except for the SIX1 p.Leu103Profs*51 variant due to its loss of nuclear translocation (Fig. 7a). Using the nucleus fractions, we then performed the DNA binding affinity assay with biotin-ssDNA. Despite their interaction with EYA1, as evidenced by the protein–protein interaction assay, all mutants exhibited obvious but varying degrees of reduction in DNA binding affinity (Fig. 7b). However, the p.Glu125Lys and p.Tyr129_Cys130del mutants did not display significant differences when compared to the wild-type, suggesting a substantial preservation of their molecular functions in the context of DNA binding affinity. The functional assay correspond with the milder clinical phenotypes observed in patients affected by these two variants (p.Glu125Lys and p.Tyr129_Cys130del), including those with DFNA23.of affected patients with these two variants (p.Glu125Lys and p.Tyr129_Cys130del) appear to be relatively mild, including DFNA23.

### Transcriptional activity

**Figure 8:**
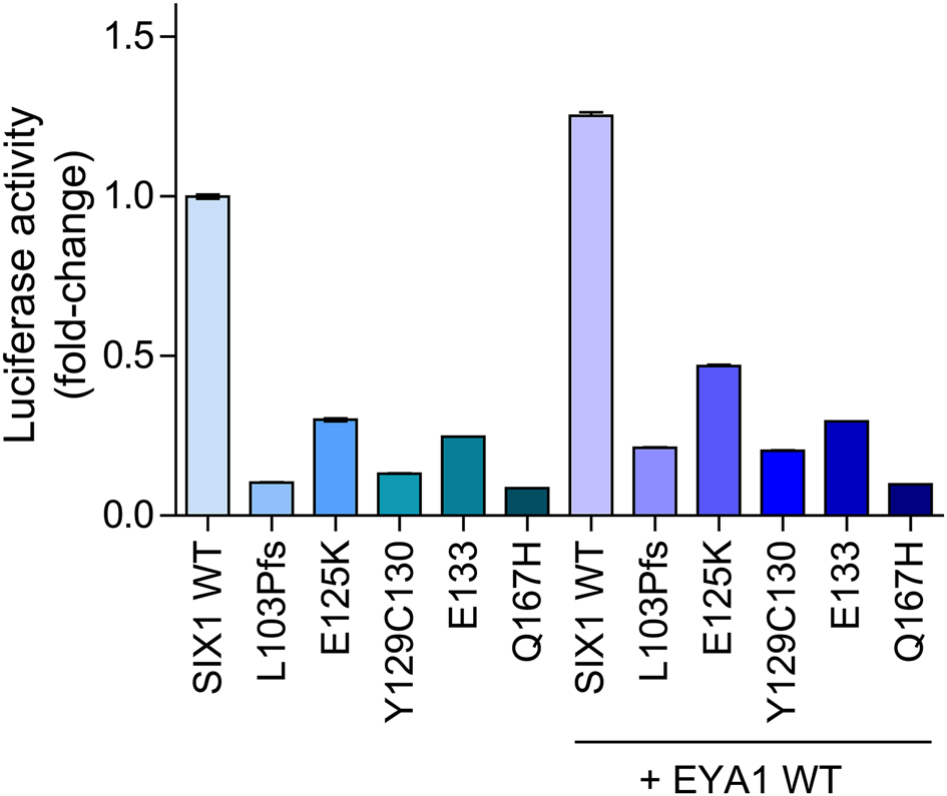
Transcriptional activity based on the luciferase reporter assay. The transcriptional efficiency of SIX1 wild-type in transfected HEK293T cells was highest using the luciferase vector system (pGL4.12[luc2CP], 2 µg). All SIX1 mutants significantly reduce the transcriptional activity required to regulate downstream target gene expression, even the EYA1 co-transfected mutants. The significance is higher in the MYOG Luc-vector than in the Single Luc-vector. SIX1 wild-type enhanced luciferase activity by approximately four-fold. In contrast, SIX1 mutants enhanced luciferase activity by less than two-fold, demonstrating a significantly poorer ability to induce transcription than the SIX1 wild-type.

We used the myogenin luciferase reporter, pGL4.12[luc2CP]-MYOG-6xMEF3, expressed alone and in conjunction with EYA1 wild-type, to assess the effects of the *SIX1* variants on transcriptional activity. To minimize the ceiling effect, we identified the luciferase system with the highest efficiency for SIX1 wild-type transcriptional activity (Fig. S3). We compared luciferase activity between the mutants and wild-type under the same conditions (i.e., MYOG-6xMEF3-luc, 0.25 µg). HEK293T cells transfected with SIX1 wild-type alone resulted in a sevenfold increase in luciferase activity compared to the internal control. In contrast, all mutants led to significant decreases in luciferase activity compared to the wild type: 10.4% for p.Leu103Profs*51, 30.0% for p.Glu125Lys, 13.2% for p.Tyr129_Cys130del, 8.6% for p.Gln167His, and 24.7% for p.Glu133del. The SIX1 wild-type could synergistically activate luciferase activity when co-expressed with EYA1, resulting in a 1.3-fold increase in luciferase activity. In contrast, co-expression of SIX1 mutants with EYA1 wild-type resulted in significant decreases in luciferase activity compared to the wild-type, by 17.0% for p.Leu103Profs*51, 37.4% for p.Glu125Lys, 16.3% for p.Tyr129_Cys130del, 7.8% for p.Gln167His, and 23.6% for p.Glu133del (Fig. 8). The transcriptional activity of the p.Leu103Profs*51 mutant was the most severely decreased, whereas the transcriptional activity of the p.Glu125Lys variant was best maintained. These results were consistent across two independent experiments conducted in triplicate.

## Discussion

This study is the first to report *SIX1* variants and expands the genotypic and phenotypic spectrum of *SIX1*-associated DFNA23 and atypical BO syndrome in South Korea. There was a lower incidence of major and minor criteria, except hearing loss, among individuals with *SIX1* variants compared to clinical *EYA1* variant cohorts, and the phenotypic features related to DFNA23 or atypical BO syndrome were highlighted in *SIX1* variants. This study’s functional findings clarify the molecular aspects of *SIX1* variants according to the SIX1–EYA1–DNA complex.

The molecular mechanisms of *SIX1* variants involve transcriptional activity, such as EYA1–SIX1 interaction, nuclear translocation, and DNA-binding affinity. SIX1 operates in concert with EYA1, a cofactor and primary cause of BOR/BO syndrome when mutated. The interaction between SIX1 and EYA1 is widely regarded as essential for the transcriptional activity of SIX1^8^, as demonstrated by the interaction between the SIX1 mutants (p.Arg110Trp, p.Tyr129Cys, and p.Glu133del) and EYA1 wild-type was inhibited in a yeast-two-hybridization assay. However, the interaction between SIX1 and EYA2 ED is unchanged in the same SIX1 mutants (p.Arg110Trp, p.Tyr129Cys, and p.Glu133del) in *Escherichia coli*^22^. In support of this finding, a size-exclusion chromatography-based gel filtration profile revealed the intact formation of an EYA1-SIX1 complex between the purified SIX1 mutant proteins^22^. Considering the conflicting results regarding the SIX1–EYA1 interaction in two distinct species, supporting evidence of protein–protein interactions using human-derived cell lines is essential. Therefore, we analyzed the functional interaction between SIX1 and EYA1 through a protein pull-down assay and co-immunostaining assays in mammalian cells. The results indicated that the SIX1 p.Leu103Profs*51 mutant, which affects the SD α6 and HD domains, failed to translocate to the nucleus even in the presence of EYA1 wild-type, suggesting that the truncated mutant (p.Leu103Profs*51) disrupts the EYA1–SIX1 complex or leads to the loss of nuclear translocation capacity due to the absence of an HD domain. Following the His-EYA1 pull-down assay, we compared the EYA1*-*SIX1 mutant (p.Leu103Profs*51) interactions to the wild-type protein, which suggested that the pathogenic mechanism in the SIX p.Leu103Profs*51 mutant could be due to the loss of nuclear translocation capacity and the absence of an HD domain. SIX1 does not possess a conventional nuclear localization signal (NLS) motif and instead relies on its HD domain^42^. In this study, the four SIX1 mutants, caused by missense variants or in-frame deletions, located in the HD domain could localize to the nucleus, either when expressed alone or in conjunction with EYA1 wild-type, suggesting that these HD domain residues (p.Glu125, p.Tyr129, p.Cys130, p.Glu133, and p.Gln167) are essential for nuclear translocation. Besides, these mutants also displayed protein–protein interactions comparable to the wild-type protein. This is in line with the structure analysis using the SIX1–EYA2ED complex, demonstrating that the protein–protein interaction is primarily mediated by a single helix (α1) of the SIX1 SD domain that fits into a binding groove on EYA2ED^32^. Based on the EYA1–SIX1 interaction assays, we suggest that the diminished transcriptional activity observed in the SIX1 mutants is attributable to reduced DNA-binding affinity.

The proposed mechanism for binding SIX1 to DNA, as suggested by the crystal structure of the human SIX1–EYA2 complex, highlights the significance of the HD domain and the α6 in the SD domain to the maintenance of SIX1-DNA binding ability^32^. Biochemical assays verified the SIX1–DNA mechanism^8,22,43^, demonstrating that BOR syndrome mutants in these residues significantly reduce DNA binding affinity. The 3D modeling and structure analysis in the present study also showed that substitution of the glutamic acid residue 167 with histidine (p.Gln167His) located in the HD domain possibly resulted in expulsion from the DNA binding cleft and loss of the SIX1–DNA interaction (see Fig. 3). This structure was confirmed by biochemical assays showing a significant decrease in DNA-binding affinity compared to the wild-type. Theoretically, the SIX1 HD domain possesses a unique characteristic among homeobox proteins (Fig. S4). The HD domain typically includes a basic loop (p.Arg110-p.Lys114 in the case of SIX1) followed by three tandem helices. The basic loop, consisting of basic residues such as arginine and lysine, is responsible for interaction with the DNA duplex. Unlike most homeobox proteins where the basic loop and the first helix are directly connected with a small amino acid gap, the SIX1 HD domain possesses a gap of over 12 amino acids between the end of the basic loop (p.Lys114) and the beginning of the first helix (p.Phe131). Although previous structural simulation studies showed that the two mutants (p.Glu125Lys and p.Tyr129His) led to alterations in structural and electrostatic potentials^44,45^, these two residues were unidentified regions in a structural basis, necessitating further characterization of the molecular architecture of the distinct junction in SIX1. Indeed, the mutants in the SIX1 HD domain previously reported exhibit reduced DNA-binding affinity^8^, which aligns with this study, indicating that all mutants exhibited obvious but varying degrees of reduction in DNA binding affinity. Specifically, both mutants (p.Glu125Lys and p.Tyr129_Cys130del) possess substantial SIX–DNA binding ability, comparable to the wild-type and correlating with the transcriptional activity. The results suggest that the molecular functions of both mutants (p.Glu125Lys and p.Tyr129_Cys130del) are partially maintained, which translates to milder DFNA23 phenotypes. Supporting this, the variants p.Glu125Lys and p.Tyr129His, with the same residue changes, manifest only DFNA23^44–46^. We believe that further elucidation of the unidentified regions in SIX1, alongside validation of the biochemical investigations, will support our findings.

The comparative study demonstrated that all *SIX1* variants led to DFNA23 or atypical BO syndrome, while only 13.3% of *EYA1* variants did. More specifically, we demonstrated that most of the criteria, including branchial and external ear anomalies, preauricular pits, and renal, middle ear, and inner ear anomalies, were significantly less frequent in *SIX1* than in *EYA1* (Fig. 2). Previous research also showed a higher prevalence of atypical BOR/BO syndrome caused by *SIX1* variants compared to *EYA1* variants^4,11^. Consequently, our results have significant clinical implications, suggesting *SIX1* as a potential candidate gene for individuals with non-syndromic hearing loss (DFNA23) and/or preauricular fistula, even without the typical BOR/BO syndrome features. Furthermore, understanding the phenotypic characteristics of affected individuals with *SIX1* variants would help mitigate the diagnostic issues and provide valuable insights into prognosis.

Given that the SIX1 and EYA1 transcription factors form a bipartite complex to regulate the development of several organs^41,47^ and act as an interactive unit, the underlying molecular mechanisms through which *SIX1* variants can manifest relatively mild phenotypes remain elusive. It is assumed that the phenotypic variability is influenced by intermolecular networks and regulatory factors that modulate the expression and transcriptional activity of the genes involved and the participation of specific cellular signaling pathways. The EYA1–SIX1–DNA complex and its binding partners, such as Sobp^17^ and Mcrs1^48^, which comprise interactive networks with SIX1 and EYA1, provide insights into the molecular mechanisms and complexity of phenotypes in the spectrum of BOR/BO syndrome. Sobp is a good example of this complexity; it is essential for the transcriptional activity of Six1 because it binds to Six1 and translocates to the nucleus with it^17^. Sobp variants act as transcriptional factor repressors that bind to Six1 and inhibit its transcriptional activity on Six1 target genes. Six1 can interact with other cytoplasmic proteins such as Sobp and is not limited to Eya factors^18^, which potentially contributes to the phenotypic variability. The association of DFNA23 and atypical BO syndrome with *SIX1* variants may indicate that SIX1 has functional redundancy during human branchial arch embryogenesis compared to EYA1. This may be due to intricate intermolecular networks involving SIX1’s binding partners, which can act as repressors of its transcriptional activity. Alternatively, the underlying mechanisms of SIX1 mutants located in the HD domain may be associated with reduced DNA-binding affinity or impaired nuclear localization rather than protein–protein interactions with EYA1. We infer that the milder phenotypes observed in individuals with *SIX1* variants, as opposed to *EYA1* variants, may be attributed to a lower reliance of SIX1 mutants on the EYA1–SIX1 complex on the regulation of transcriptional activity. Interestingly, most *SIX1* variants located in the HD domain are linked to DFNA23 in the literature^45^, consistent with this study’s findings. Indeed, it has been established that the SIX1 mutants under investigation interact effectively with its cofactor EYA1.

## Supplementary Information

Supplementary Information.

## Data Availability

Upon reasonable request, the corresponding author can provide access to the datasets analyzed in the current study. Supplementary information includes uncropped western blots for each figure.
