# Supplementary material for: Phenotypic and molecular basis of SIX1 variants linked to non-syndromic deafness and atypical branchio-otic syndrome in South Korea
Source: Sci Rep. 2023 Jul 21;13:11776. doi: 10.1038/s41598-023-38909-w (PMC10361970; doi:10.1038/s41598-023-38909-w)
Supplement: Supplementary file 1 — Supplementary Information. [file 41598_2023_38909_MOESM1_ESM.pdf]

Scientific Reports

Supplementary Information

Phenotypic and molecular basis of *SIX1* variants linked to non-syndromic deafness and atypical branchio-otic syndrome in South Korea

Somin Lee[1#], Yejin Yun[1#], Ju Hyuen CHA[1], Jin Hee Han[2], Dae Hee Lee[3], Jae-Jin Song[2], Moo Kyun Park[1], Jun Ho Lee[1], Seung Ha Oh[1], Byung Yoon Choi[2]*, and Sang-Yeon Lee[1,4,5]*

[1]Department of Otorhinolaryngology-Head and Neck Surgery, Seoul National University Hospital, Seoul National University College of Medicine, Seoul, South Korea

[2]Department of Otorhinolaryngology-Head and Neck Surgery, Seoul National University Bundang Hospital, South Korea

[3]CTCELLS, Inc., 21, Yuseong-daero, 1205beon-gil, Yuseong-gu, Daejeon, Rep.of Korea

[4]Department of Genomic Medicine, Seoul National University Hospital, Seoul, South Korea

[5]Sensory Organ Research Institute, Seoul National University Medical Research Center

#These authors contributed equally to this work.

*These authors supervised equally to this work.

Corresponding author
Sang-Yeon Lee
Department of Otorhinolaryngology-Head and Neck Surgery, Seoul National University College of Medicine
Department of Genomic Medicine, Precision Medicine & Rare Disease Center
Seoul National University Hospital, Jongno-Gu, Daehak-Ro, 101, Seoul, Korea
Tel: +82-10-3645-9715
E-mail: maru4843@hanmail.net

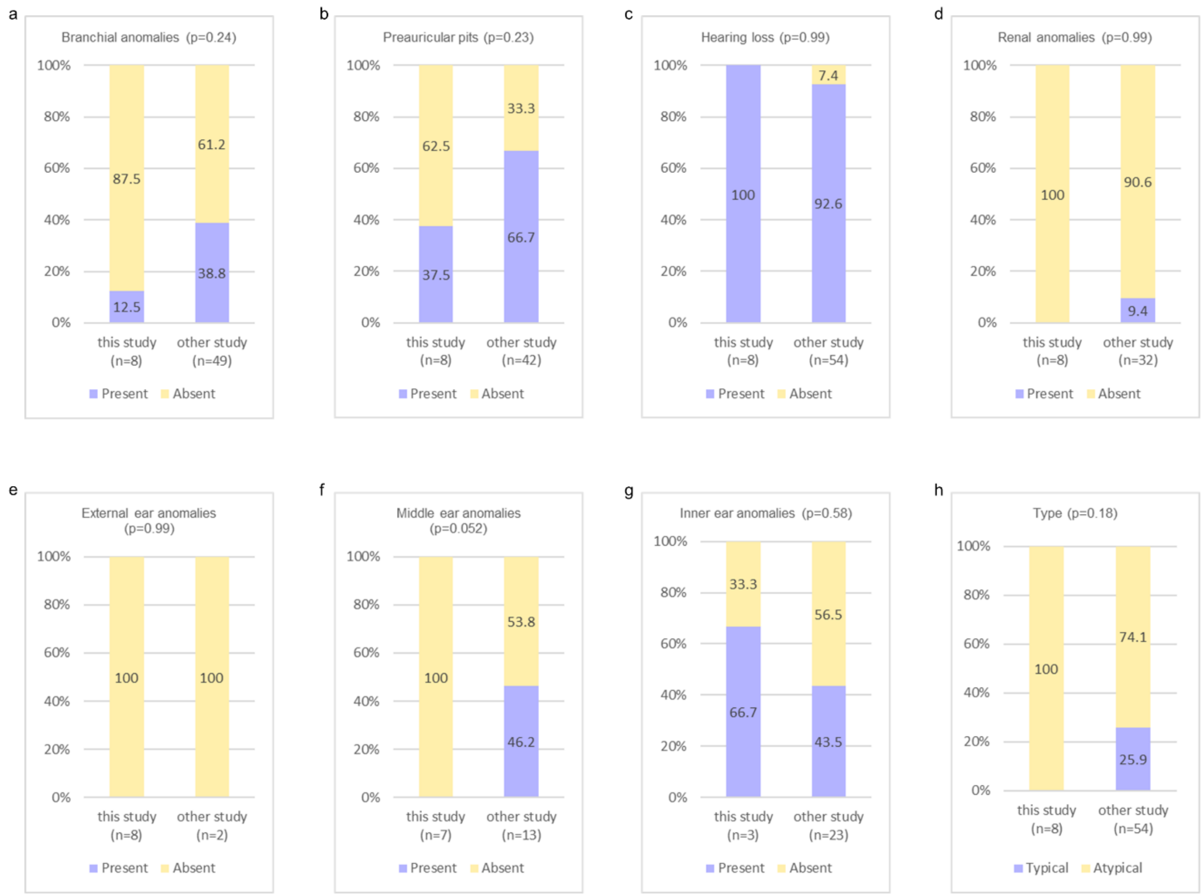

Supplementary Figure S1. Comparison of statistics for each phenotype of the *SIX1* in this study versus those in other studies. No significant difference in the overall phenotypes was observed.

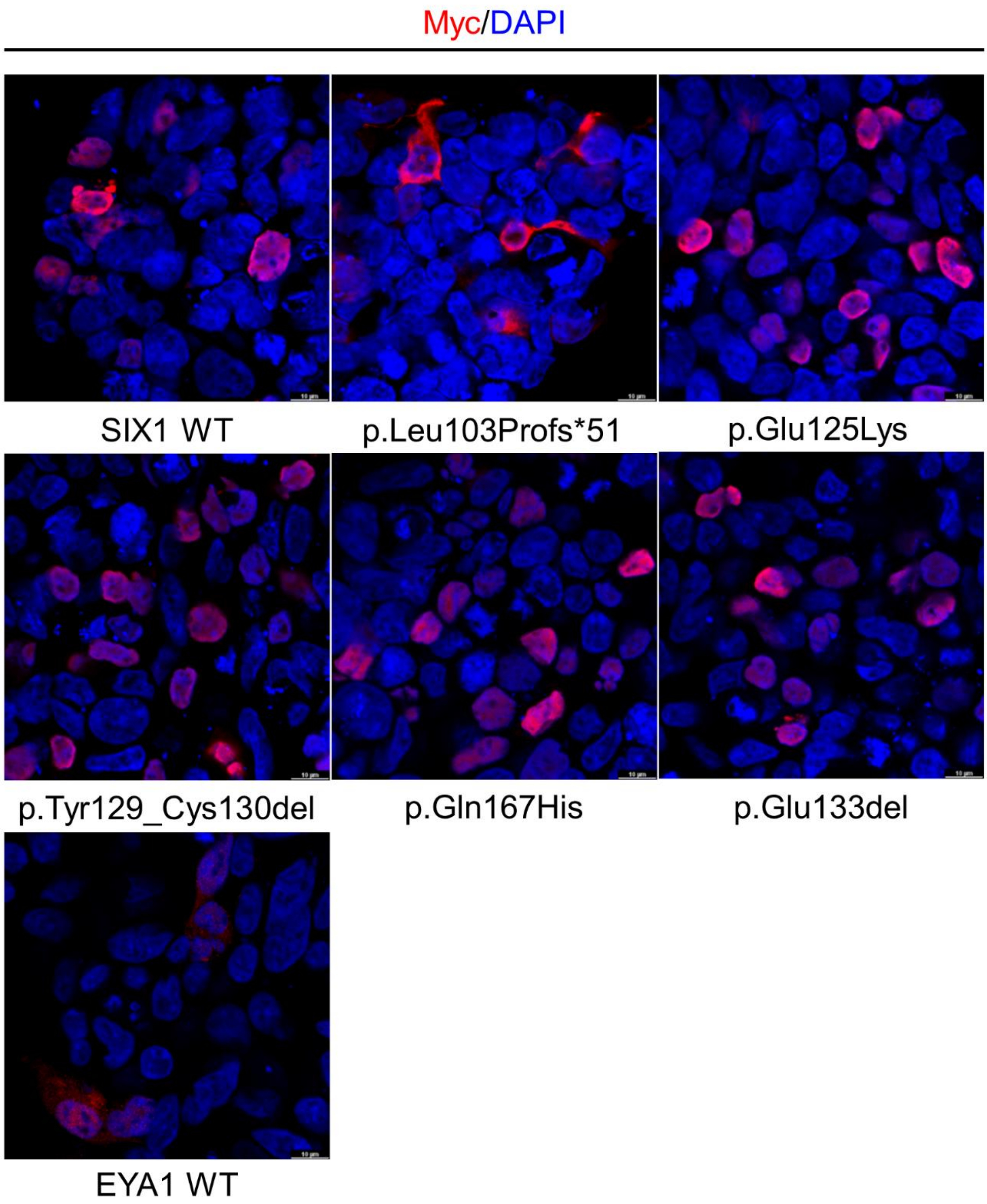

Supplementary Figure S2. Subcellular localization of SIX1 wild-type and mutants. Immunofluorescence of HEK293 cells single transfected with C-terminal Myc-DDK-tagged SIX1 wild-type, p.Leu103Pfs*51, p.Glu125Lys, p.Tyr129_Cys130del, p.Gln167His, p.Glu133del, and EYA1 wild-type. Except for the p.Leu103Profs*51, all mutant SIX1 and wild-type proteins were localized in the nucleus, and full-length EYA1 was localized in the cytoplasm.

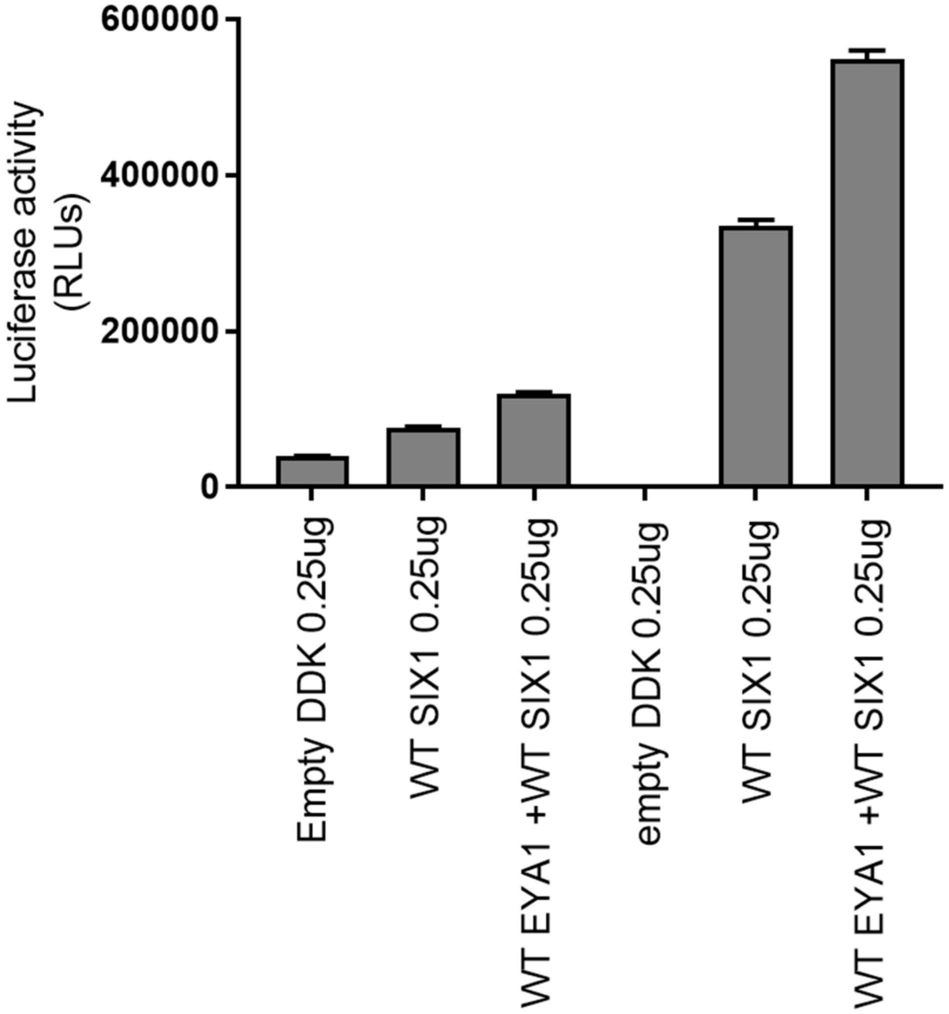

Supplementary Figure S3. Optimization of efficiency to measure the transcriptional activity of SIX1 wild-type. To minimize the ceiling effect, we used the luciferase system with the highest efficiency at a concentration of 0.25 µg of the MYOG-6xMEF3-luc plasmid.

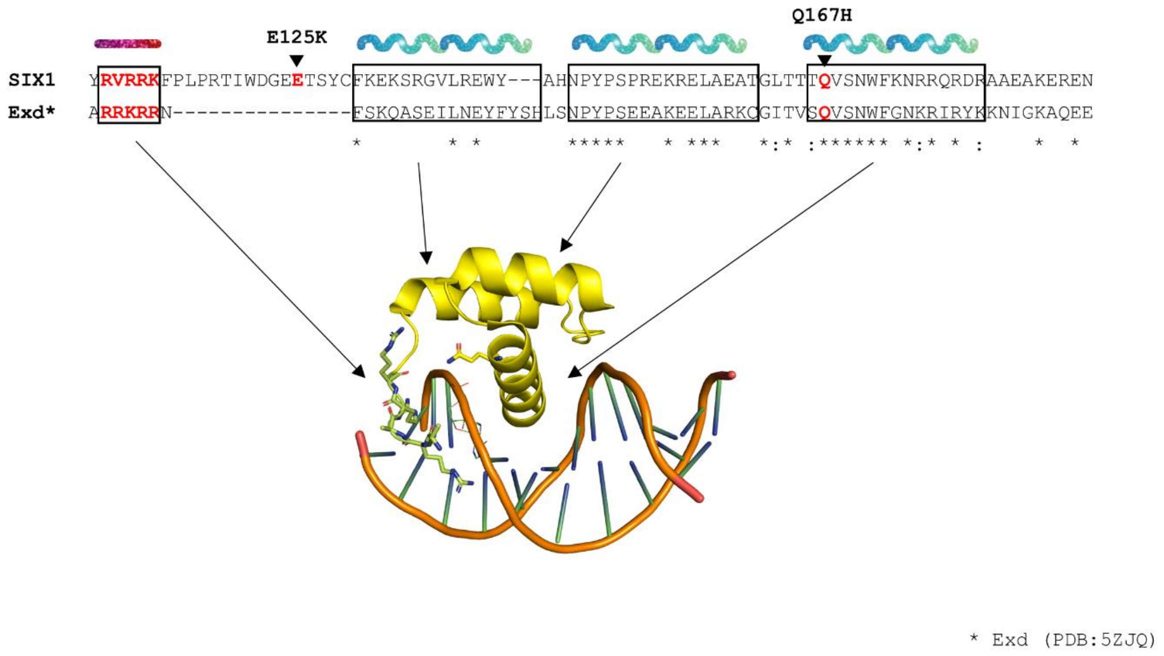

Supplementary Figure S4. Distinct structural characteristics of the SIX1 HD domain. The SIX1 HD domain is characterized by a gap of over 12 amino acids between the end of the basic loop (p.Lys114) and the beginning of the first helix (p.Phe131).

Figure 4

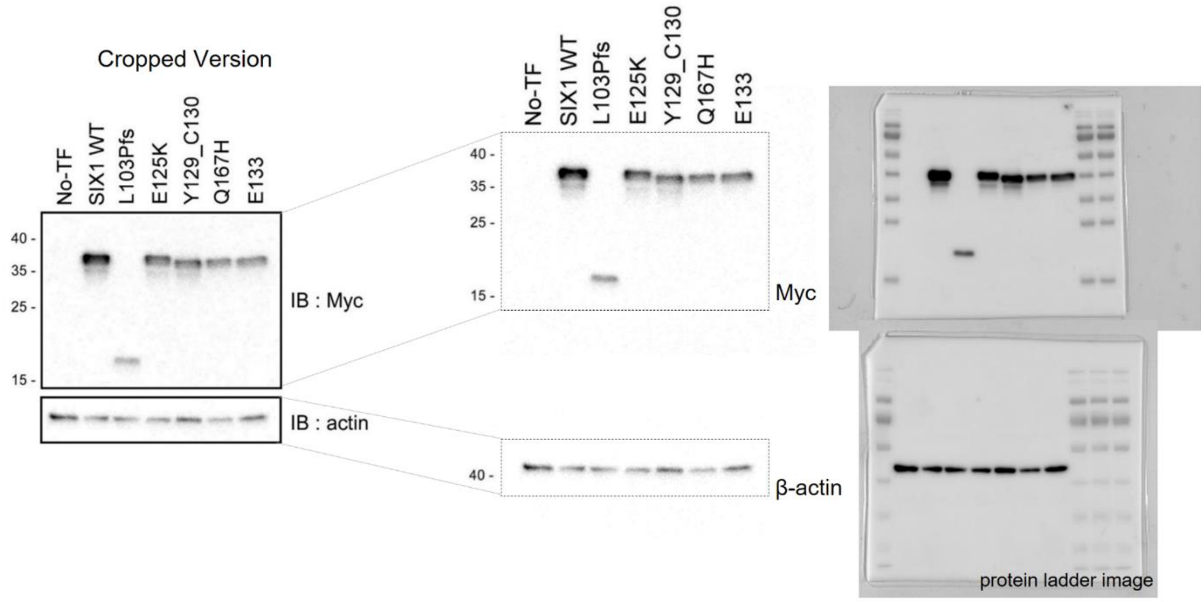

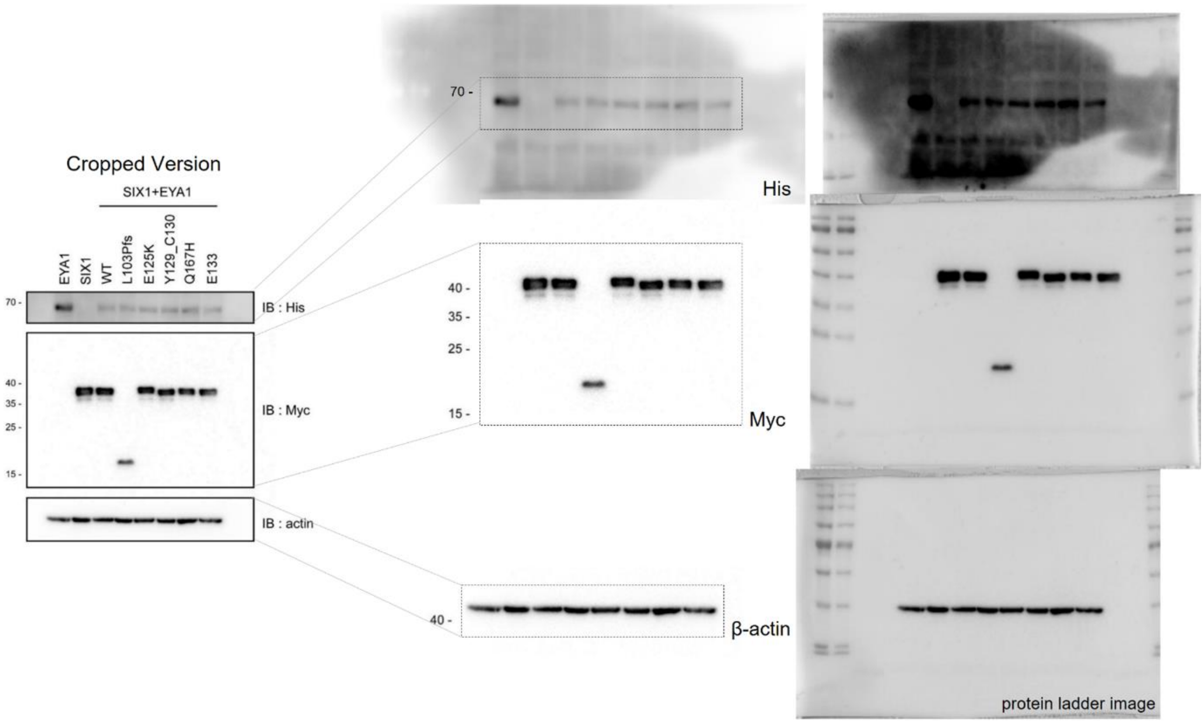

Supplementary Figure S5. Original blots of Fig. 4

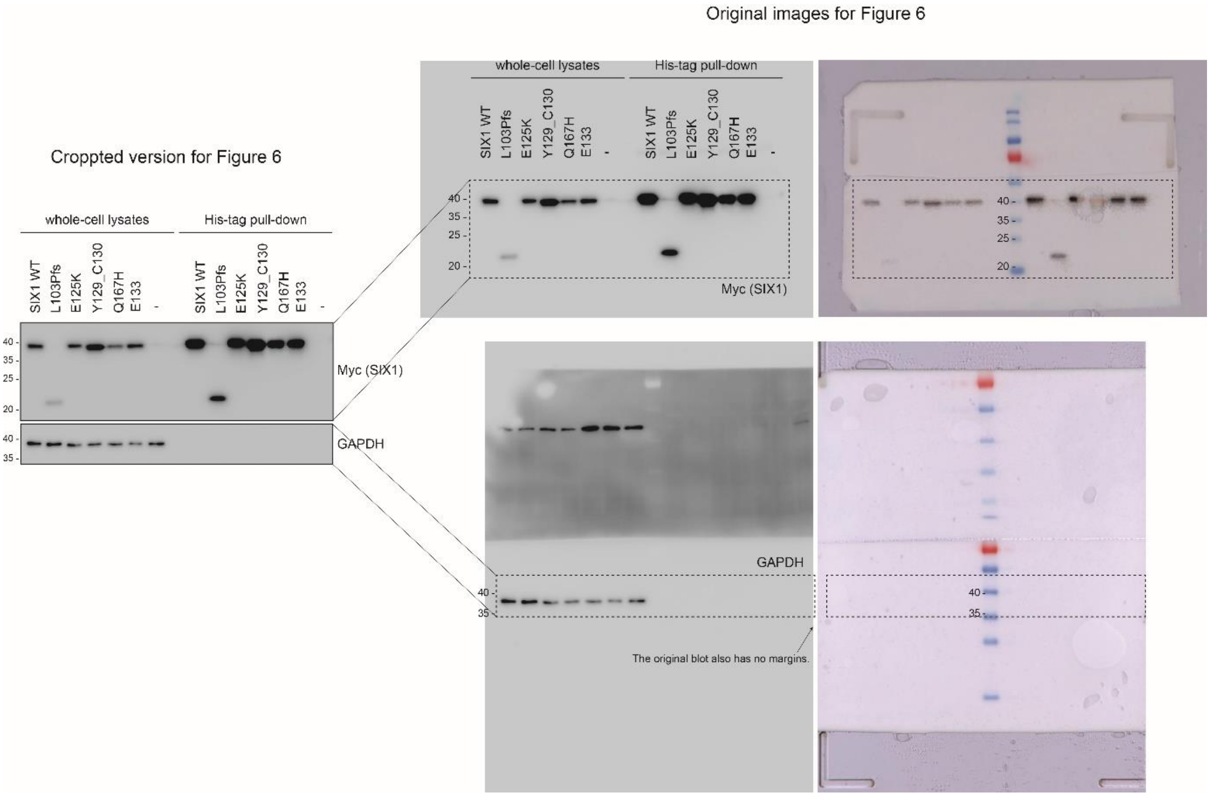

Supplementary Figure S6. Original blots of Fig. 6

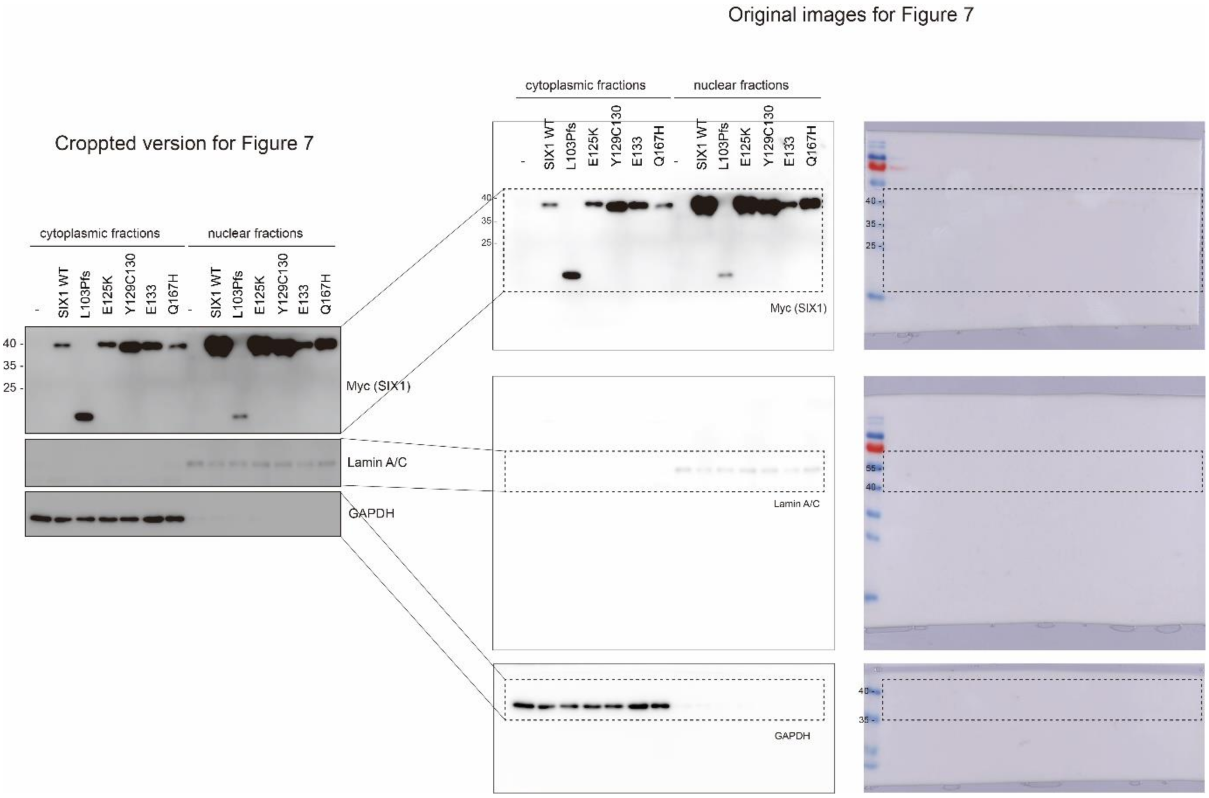

Supplementary Figure S7. Original blots of Fig. 7

Supplementary Table 1. Detailed clinical features of *SIX1* according to criteria BOR/BO syndrome.

Family ID.	Gene	Variant	Branchial anomalies	Preauricular pits	Hearing loss	Renal anomalies	External ear anomalies	Middle ear anomalies	Inner ear anomalies	Typical vs. Atypical*	Reference
22260	*SIX1*	c.50T>A:p.Val17Glu	1/1	0/1	1/1	0/1	N/A	N/A	N/A	Atypical 1/1	Kochhar et al. 2008
21870	*SIX1*	c.218A>C:p.His73Pro	2/2	0/2	1/2	0/2	N/A	N/A	N/A	Atypical 2/2	Kochhar et al. 2008
SB468-909	*SIX1*	c.307dupC:p.Leu103ProfsTer51	0/1	0/1	1/1	0/1	0/1	0/1	N/A	Atypical 1/1	this study
20050	*SIX1*	c.317T>G:p.Val106Gly	0/1	1/1	1/1	N/A	N/A	N/A	N/A	Atypical 1/1	Kochhar et al. 2008
K6/7	*SIX1*	c.328C>T:p.Arg110Trp	1/1	1/1	1/1	0/1	N/A	N/A	N/A	Typical 1/1	Ruf et al. 2004
S2120	*SIX1*	c.328C>T:p.Arg110Trp	1/1	1/1	1/1	N/A	N/A	N/A	N/A	Typical 1/1	Ruf et al. 2004
2221	*SIX1*	c.328C>T:p.Arg110Trp	8/12	12/12	12/12	1/7	N/A	6/8	8/8	Typical 9/13 Atypical 4/13	Ceruti et al. 2002
2440	*SIX1*	c.328C>T:p.Arg110Trp	N/A	N/A	1/1	N/A	N/A	N/A	N/A	Atypical 1/1	Kochhar et al. 2008
20540	*SIX1*	c.328C>T:p.Arg110Trp	1/1	0/1	1/1	N/A	N/A	N/A	N/A	Atypical 1/1	Kochhar et al. 2008
21480	*SIX1*	c.329G>A:Arg110Gln	0/1	1/1	1/1	0/1	N/A	N/A	N/A	Atypical 1/1	Kochhar et al. 2008
20800	*SIX1*	c.334C>T:p.Arg112Cys	1/1	0/1	1/1	0/1	N/A	N/A	N/A	Atypical 1/1	Kochhar et al. 2008
Family 2	*SIX1*	c.364T>A:p.Trp122Arg	1/11	5/11	14/15	0/3	N/A	0/3	2/3	Typical 1/14 Atypical 13/14	Sanggaard et al. 2007
SB940	*SIX1*	c.373G>A:p.Glu125Lys	0/2	0/2	2/2	0/2	0/2	0/2	1/1	Atypical 2/2	this study
KH	*SIX1*	c.373G>A:p.Glu125Lys	0/5	4/5	5/5	0/4	N/A	N/A	0/3	Atypical 5/5	Mosrati et al. 2011
F1038	*SIX1*	c.386A>G:p.Tyr129Cys	1/1	1/1	1/1	0/1	N/A	N/A	N/A	Typical 1/1	Ruf et al. 2004
Ito1	*SIX1*	c.386A>G:p.Tyr129Cys	0/1	1/1	1/1	0/1	N/A	N/A	N/A	Atypical 1/1	Ito et al. 2006
P-D26	*SIX1*	c.385T>C:p.Tyr129His	0/7	N/A	7/7	0/7	N/A	N/A	0/7	Atypical 7/7	Hong et al.2021
SH693	*SIX1*	c.386_391del:p.Tyr129_Cys130del	0/2	2/2	2/2	0/2	0/2	0/2	N/A	Atypical 2/2	this study
SH613	*SIX1*	c.397_399del:p.Glu133del	0/1	1/1	1/1	0/1	0/1	0/1	1/1	Atypical 1/1	this study

F1120	*SIX1*	c.397_399del:p.Glu133del	1/1	0/1	1/1	1/1	N/A	N/A	N/A	Typical 1/1	Ruf et al. 2004
SH529	*SIX1*	c.501G>C:p.Gln167His	1/2	0/2	2/2	0/2	0/2	0/1	0/1	Atypical 2/2	this study
1226	*SIX1*	c.560+3A>T	1/1	1/1	0/1	0/1	0/1	0/1	0/1	Atypical 1/1	Krug et al. 2010
162	*SIX1*	c.746C>T:p.Pro249Leu	0/1	0/1	0/1	1/1	0/1	0/1	0/1	Atypical 1/1	Krug et al. 2010

SIX1, canonical transcript NM_005982.4; Abbreviations: N/A, not available.

*Note, If the observed symptoms do not match the predefined requirements for a diagnosis of BOR/BO syndrome, specifically either at least three major criteria or a combination of two major and two minor ones, then the condition can be classified as a atypical BOR/BO syndrome.
